# Heterotopic Pregnancy - A Diagnostic Challenge. Six Case Reports and Literature Review

**DOI:** 10.7759/cureus.6080

**Published:** 2019-11-05

**Authors:** Usman Nabi, Amman Yousaf, Fariha Ghaffar, Sadia Sajid, Mohamed Mohamed Helmi Ahmed

**Affiliations:** 1 Radiology, Hamad Medical Corporation, Doha, QAT; 2 Radiology, Hamad General Hospital, Doha, QAT; 3 Internal Medicine, Allama Iqbal Medical College, Lahore, PAK

**Keywords:** ectopic pregnancy, heterotopic pregnancy, assisted reproductive techniques, tubal pathologies, transvaginal ultrasound, laparoscopic salpingectomy

## Abstract

Heterotopic pregnancy is defined as the simultaneous presence of intrauterine and ectopic pregnancies. It is a rare condition, but due to the increasing use of artificial reproductive techniques, the incidence of heterotopic pregnancy is increasing. Most of the patients with heterotopic pregnancy have a previous history of infertility or tubal diseases. In this case series, we are presenting six cases of heterotopic pregnancy. Three of them had a history of assisted reproductive technique: one patient had in vitro fertilization with three embryos transferred, and two patients received follicular stimulating hormone therapy. In one of the cases, heterotopic pregnancy was missed on an initial transabdominal scan, and in the following weeks, it was diagnosed on transvaginal ultrasound. Five patients underwent laparoscopic salpingectomy, and one patient had laparotomy and then a salpingectomy was done. Follow-up ultrasound scans for intrauterine pregnancy (IUP) showed abortion of the IUP, except in one patient who delivered a healthy full-term baby via spontaneous vaginal delivery. Therefore, there is a need to develop diagnostic criteria to rule out heterotopic pregnancy if the patient underwent any type of assisted reproductive techniques. We are emphasizing the need for more careful scanning of the adnexa via transvaginal ultrasound, especially in high-risk patients, even if the intrauterine gestation is confirmed.

## Introduction

The coexistence of intrauterine pregnancy and ectopic pregnancy is called heterotopic (HT) pregnancy. The incidence of ectopic pregnancy is 0.006% - 0.001% in spontaneous cases, while in assisted reproductive technique (ART), it is 1% - 3% [1]. In 1972, first heterotopic pregnancy after in vitro fertilization was reported [2]. It is a sporadic condition but is becoming more common after ART. The most common ectopic site is the fallopian tube, both in spontaneous and ART heterotopic pregnancies. The cornual site is the second most common site, while HT in the cervix, ovary, and abdomen is extremely rare. Almost 60% - 70% of HT cases result in live childbirth with outcomes similar to that of singleton pregnancies. A delayed diagnosis can result in increased rates of morbidity and mortality both for the mother and intrauterine gestation [1, 3].

HT pregnancy could be asymptomatic in 24% of cases, can cause abdominal pain in 72%, and 54% present with vaginal bleeding [4]. In HT pregnancy, the chances of abortion are doubled. Most of the literature are case reports emphasizing on early TVU after ART for early diagnosing of an HT pregnancy to avoid life-threatening complications.

Herein, we present six cases of HT pregnancy. Three had a history of ART, and three conceived spontaneously.

## Case presentation

Case 1

**Figure 1:**
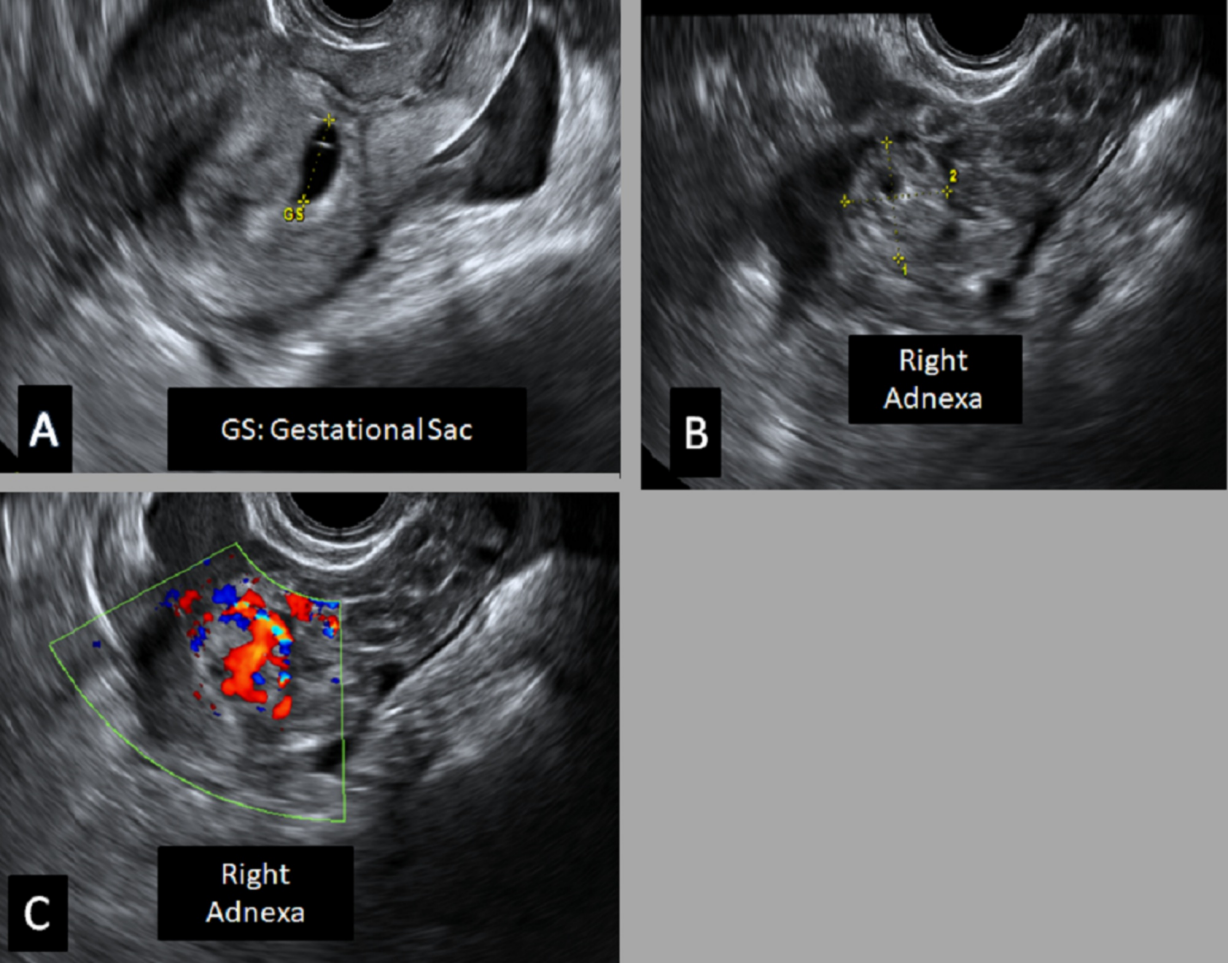
Transvaginal ultrasound A) Intrauterine elongated gestational sac (GS); B-C) There is a right adnexal complex lesion containing rounded thick-walled echogenic structure; Doppler study revealed a ring of fire appearance, likely a picture of right adnexal ectopic pregnancy with hematoma favoring the diagnosis of heterotopic pregnancy.

A 26-year-old female, with a history of primary infertility, underwent follicular stimulating hormone therapy. On follow-up, she had a positive serum beta-human chorionic gonadotropin (hCG) test. On transvaginal ultrasound, an intrauterine gestational sac of 3 mm was noted (corresponding to the gestational age of approximately four weeks). After four weeks, the patient presented with right lower quadrant abdominal pain. She was hemodynamically stable. On transvaginal ultrasound, a right adnexal complex lesion (echogenic ring sign) was noted, in addition to the intrauterine gestational sac. On the Doppler study, the ring of fire sign was noted, suggesting ruptured ectopic pregnancy (Figure 1).

Thus, the diagnosis of heterotopic pregnancy was established. The patient underwent laparoscopic right total salpingectomy. Histopathology examination confirmed the diagnosis of an ectopic pregnancy. The patient underwent repeat ultrasound after one week that showed no fetal pole and confirmed missed abortion of intrauterine pregnancy (IUP).

Case 2

**Figure 2:**
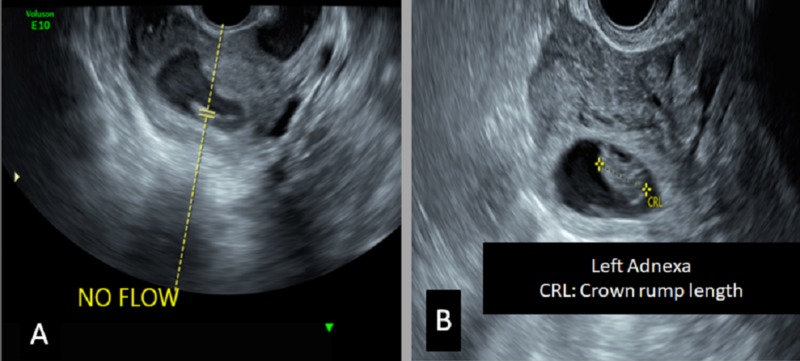
Transvaginal ultrasound A) A single nonviable intrauterine gestational sac is noted; B) There is evidence of the gestational sac hosting fetal pole in the left adnexa as well. These findings confirm the diagnosis of heterotopic pregnancy.

A 37-year-old female (G3P0A2) with a history of an ectopic pregnancy managed conservatively presented in the emergency room with lower abdominal pain. On examination, the patient's vitals were stable, and there was tenderness in the left lower abdomen. Transvaginal ultrasound showed a non-viable IUP and a viable left adnexal ectopic pregnancy (Figure 2).

These findings confirmed the diagnosis of heterotopic pregnancy. A left salpingectomy was done via a mini-laparotomy for the ectopic conceptus, and suction evacuation of the uterus was done as well.

Case 3

**Figure 3:**
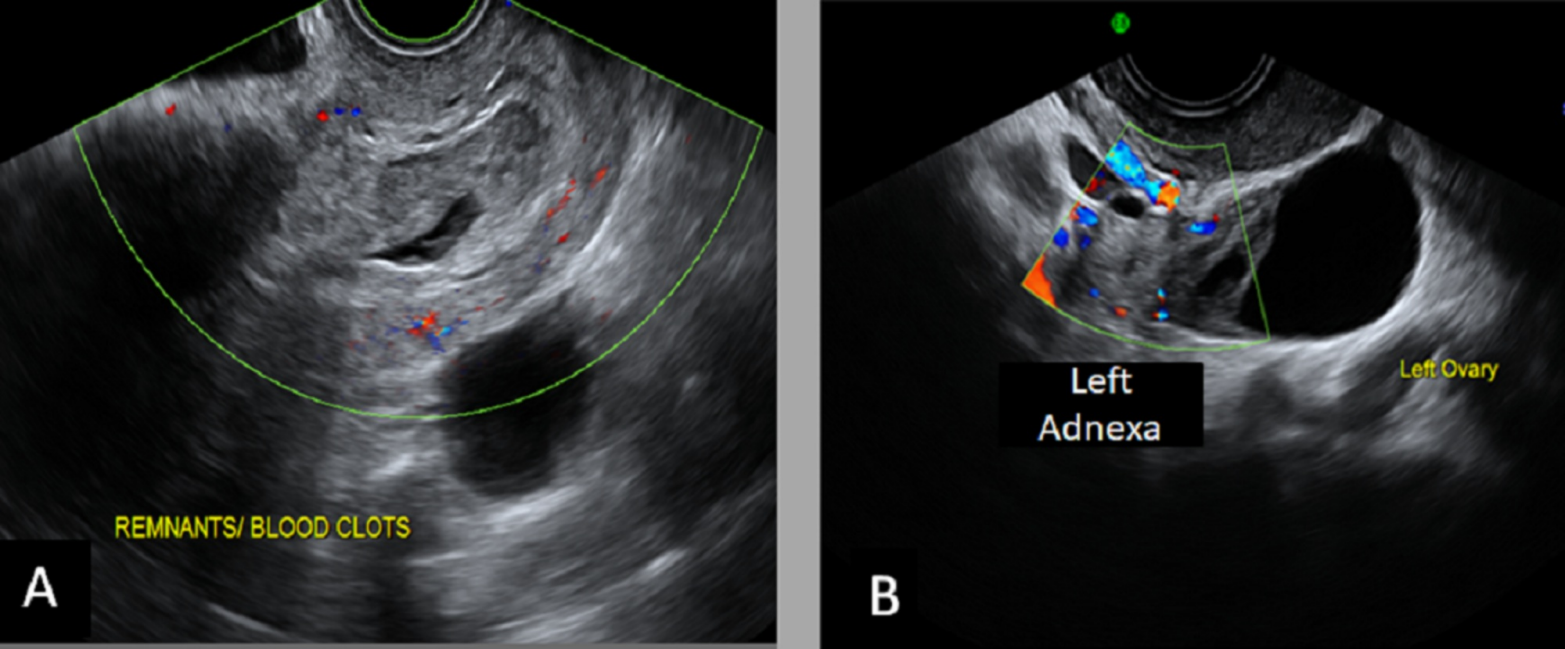
Transvaginal ultrasound A) There is an elongated sac-like structure seen in the lower uterine segment with a surrounding echogenic area (hematoma); B) A left adnexal echogenic mass is seen with peripheral vascularity noted by a color Doppler study. These findings are in favor of heterotopic pregnancy with the incomplete abortion of the intrauterine pregnancy.

A 43-year-old female (G8P4A3) presented to the emergency department with mild vaginal bleeding and lower abdominal pain. On examination, the patient was alert and vitally stable. High-resolution transvaginal ultrasound (US) confirmed an incomplete abortion. On color Doppler, the ring of fire sign was noted in the left adnexa (Figure 3).

These findings suggested the diagnosis of heterotopic pregnancy. The patient underwent suction and evacuation with laparoscopic left salpingectomy on the same admission. On follow-up labs, the beta-hCG showed a significant downward trend.

Case 4

**Figure 4:**
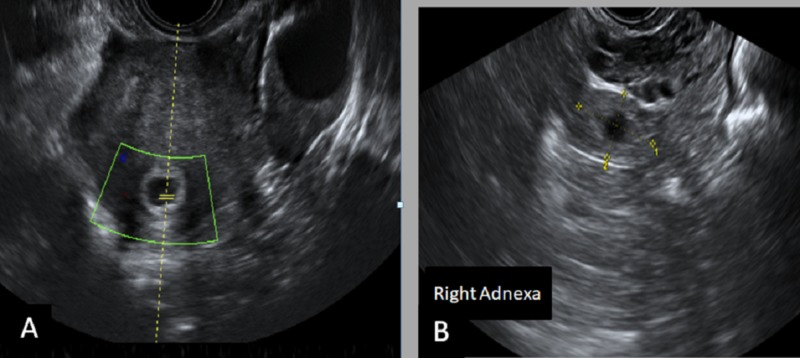
Transvaginal ultrasound A) There is a single nonviable intrauterine gestational sac; B) A rounded echogenic ring-like right adnexal lesion is noted as well. The diagnosis is in favor of heterotopic pregnancy.

A 40-year-old female (G4P2A1) with a history of secondary infertility underwent ovulation induction treatment (follicle-stimulating hormone injections). She presented in the emergency department with lower abdominal pain. On examination, her vitals were stable with mild abdominal tenderness. Her beta-hCG was positive. Transvaginal ultrasound showed a non-viable intrauterine pregnancy and right adnexal ectopic pregnancy (Figure 4).

These findings were suggestive of heterotopic pregnancy. Laparoscopic right salpingectomy was performed, and follow-up beta-hCG showed a downward trend.

Case 5

**Figure 5:**
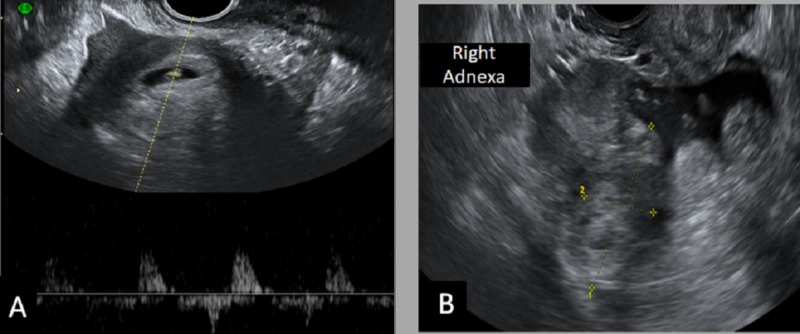
Transvaginal ultrasound A) The viable intrauterine gestational sac is seen with the fetal heartbeat; B) a right adnexal mass lesion with surrounding hematoma is noted as well, indicative of heterotopic pregnancy.

A 30-year-old female (G2P1A0) with no significant past medical history presented to the emergency department with abdominal pain. On examination, her vitals were stable. Her beta-hCG was positive. Transvaginal ultrasound showed a viable intrauterine pregnancy and an ectopic pregnancy in the right adnexa with a possible surrounding hematoma (Figure 5).

A laparoscopic right salpingectomy was done for this patient. Regular obstetric follow-up was done for the intrauterine pregnancy, and at term, the baby was delivered via spontaneous vaginal delivery with no significant complications.

Case 6

**Figure 6:**
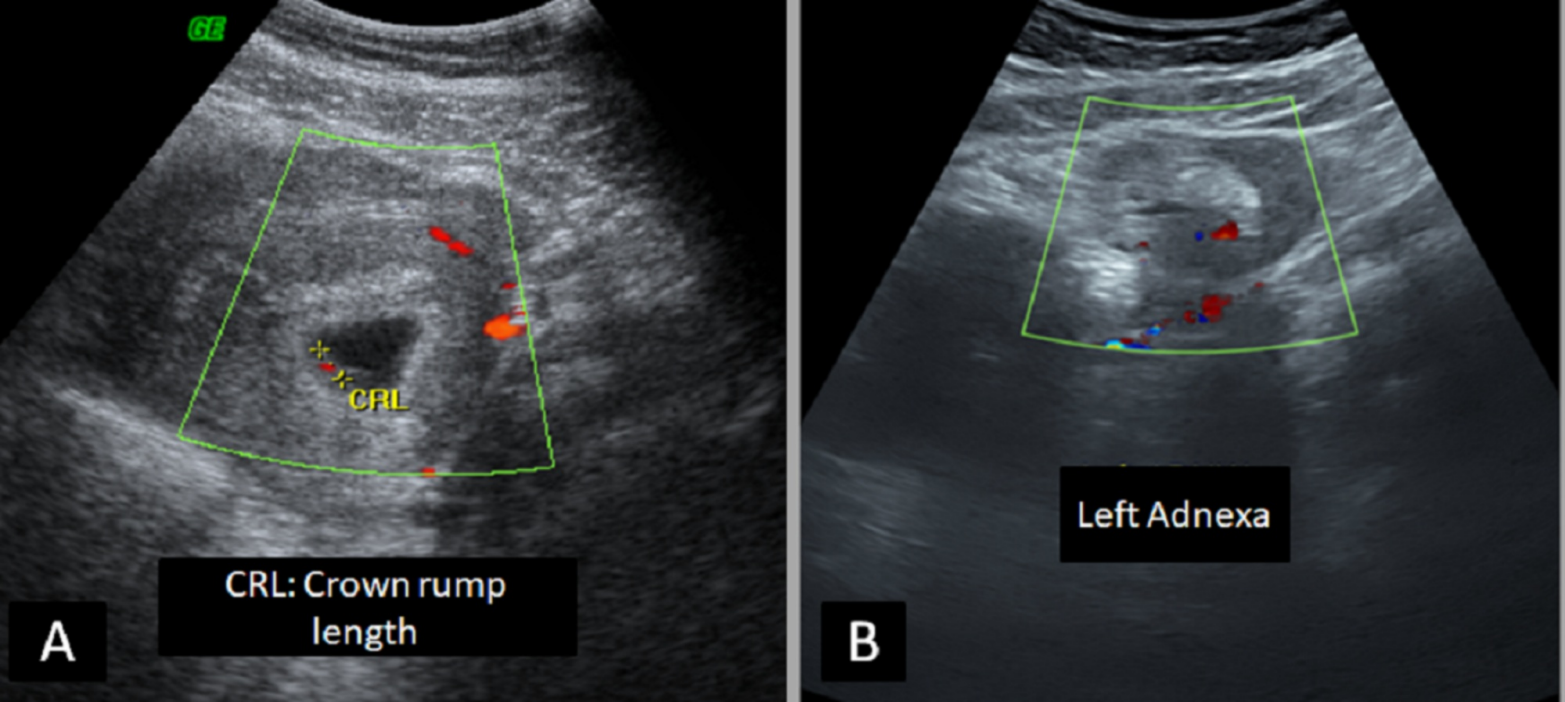
Transvaginal ultrasound A) There is a single viable intrauterine gestational sac; B) a large hematoma with a central, thick echogenic ring is seen, indicative of ruptured left ectopic pregnancy. A right adnexal lesion is also noted. These findings favor the diagnosis of a disturbed heterotopic pregnancy.

A 30-year-old female (G2P0A1) with a history of in vitro fertilization (three embryos transferred) presented with lower abdominal pain, vomiting, and vaginal discharge. On examination, she was hemodynamically stable. Transvaginal ultrasound showed a viable intrauterine pregnancy and a left adnexal ectopic pregnancy with surrounding hematoma suggestive heterotopic pregnancy (Figure 6).

A laparoscopic left salpingectomy was done, and the intraoperative findings confirmed ruptured ectopic. On follow-up ultrasound, an incomplete abortion of the IUP was noted.

## Discussion

Infertility affects 8% - 12% of people worldwide. Advances in the treatment of infertility in conjunction with causes of infertility can result in multiple, ectopic, and HT pregnancies [4]. HT pregnancy is the presence of two gestations, one intrauterine with one or more extrauterine pregnancies. The usual sites of extrauterine pregnancy are the fallopian tubes, ovaries, pelvis, and very rarely, the abdomen [6-7].

HT pregnancy can occur spontaneously or after ART and ovulation induction. It is very rare without a past gynecological history. The rates are 30 - 60 times higher after assisted reproduction as compared to spontaneous conception, thus making these techniques challenging to prevent unwanted life-threatening complications if not diagnosed and managed early in the course. Incidence after spontaneous reproduction is 1:10,000 to 1:30,000, while after assisted reproduction, it increases to 1:100 [8-9].

Its first description was done during an autopsy by Duverney in 1708, which was followed by its identification in ovulation induction, in vitro fertilization (IVF)-embryo transfer, as well as during gamete intrafallopian transfer [8].

It was proposed that 71% of the patients with HT pregnancy have at least one risk factor [3]. Tubal pathologies (infection, tubal surgery, previous ectopic pregnancy, sterilization) are the single most crucial risk factor for HT pregnancy. Exogenous hormones, ovarian factors, zygote abnormalities, endometriosis, unilateral salpingectomy, and pelvic abnormalities are other common factors [9]. Uterine contractions induced by the transfer catheter, direct injection of the embryo into the fallopian tube, volume of transfer, number of embryos transfer, and ovarian hyperstimulation drugs can affect the rates of heterotopic pregnancies after IVF [9].

Early diagnosis of HT is challenging because of the detection of an intrauterine (IU) implanted embryo and raised beta-hCG can mask the need to scan the adnexa in an asymptomatic patient. Double-checking of beta-hCG levels on Days 14 and 21 and studying the ratio of hCG21/hCG14 have suggested the viability of the gestation if the ratio is between 10 - 15 [10]. Simultaneous diagnosis of an HT pregnancy after ART requires more expertise and is more challenging because the enlarged size of the stimulated ovaries can mask the ectopically implanted pregnancy. Almost half of the HT pregnancies are detected during emergency laparotomies secondary to tubal ruptures. Therefore, it was recommended to do high-resolution transvaginal ultrasonography (TVUS) with close adnexal scanning at four to six weeks after embryo transfer for the possible early diagnosis of HT pregnancy to prevent unwanted complications [9]. In addition to this, TVUS was found to be better in early diagnosis as compared to transabdominal US. It detects almost 70% of cases between five to eight weeks of gestation [11-12]. In some centers, magnetic resonance imaging (MRI) is done to rule out HT pregnancy [9].

Treatment depends upon the severity of presentation/detection, site of ectopic pregnancy, number of previous pregnancies, the viability of intrauterine pregnancy, the expertise of the physicians in centers, and the socioeconomic status of the patients. Success rates for delivering a live newborn was 66%, while rest resulted in early or late miscarriages [5]. Expectant management can be practiced in patients with unruptured gestation and a decreasing trend of beta-hCG [13]. Systemic methotrexate (MTX) is avoided in the case of a viable intrauterine fetus, but in the case of abdominal HT pregnancy, MTX has been used for the remaining abdominal trophoblastic tissues [7, 9].

Usually, the least invasive procedures should be preferred for a better outcome for intrauterine pregnancy. Laparoscopy (salpingostomy, salpingectomy) is the treatment of choice because of a better outcome for IU pregnancy and the least harmful effects to the intrauterine gestation [14]. Selective embryo aspiration under ultrasound guidance is also one of the options. However, it demands expertise to do the procedure, and there is a high incidence of treatment failure, tubal rupture, delayed bleeding, and hematoma formation [9]. Potassium chloride and hyperosmolar injection into the embryo have also been reported.

## Conclusions

These case reports and literature review emphasize on focussed scanning of the adnexa in patients with ART and embryo transfer, even in asymptomatic cases. Exclusion of extrauterine gestation should be made the standard to rule out heterotopic pregnancy. In patients having higher than expected levels of serum beta-hCG but having only single intrauterine gestation, close monitoring by repeated serum beta-hCG levels and transvaginal ultrasound is advisable.
